# Depressive Symptoms and Glycemic Control Among Children and Adolescents With Diabetes: The Mediation Effect of Self-Care Behaviors

**DOI:** 10.1155/pedi/8850165

**Published:** 2025-10-23

**Authors:** Huaikai Song, Yixuan Huang, Jianqun Li, Yunyue Ding, Zhihua Luo, Mingwei Chen, Xiujing Cao

**Affiliations:** ^1^Department of Epidemiology and Biostatistics, School of Public Health, Anhui Medical University, Hefei, Anhui, China; ^2^Department of Endocrinology, The First Affiliated Hospital of Anhui Medical University, Hefei, Anhui, China; ^3^Department of Pediatrics, The First Affiliated Hospital of Anhui Medical University, Hefei, Anhui, China

**Keywords:** children and adolescents, depressive symptoms, diabetes, glycemic control, self-care behaviors

## Abstract

**Objective:**

This study aimed to assess the association between depressive symptoms and glycemic control among children and adolescents with diabetes and to determine if their self-care behaviors mediate this association.

**Methods:**

A total of 207 patients of children and adolescents with diabetes were included in a cross-sectional survey study. The Chinese version of the Children's Depression Inventory (CDI) was used to evaluate the depressive symptoms of the patients. The Chinese version of the Summary of Diabetes of Self-Care Activities (SDSCA) was used to evaluate the level of diabetes self-care behaviors. The values of HbA1c of children and adolescents with diabetes were obtained from patients' medical history cases or self-reporting. Structural equation modeling (SEM) was used to examine the mediation effect of self-care behaviors between depressive symptoms and glycemic control.

**Results:**

In 207 children and adolescents with diabetes, the total score of depressive symptoms was 12.71 ± 6.73 and the total score of self-care behaviors was 42.31 ± 14.09. The HbA1c of the patients was 9.14 ± 2.55%. High depressive symptoms and low self-care behaviors are related to high levels of HbA1c (all *p*  < 0.001). The results revealed that the effect of depressive symptoms on glycemic control was partly mediated by self-care behaviors and the mediation effect accounts for 30.65% of the total effect.

**Conclusions:**

Depressive symptoms show a significant association with glycemic control among children and adolescents with diabetes, with self-care behaviors serving as a partial mediator in this relationship. Depressive severity may influence glycemic control partly by affecting self-care behaviors.

## 1. Introduction

Diabetes mellitus (DM) is one of the chronic metabolic diseases among children and adolescents, which seriously affects their physical and mental health [1]. Type 1 DM (T1DM) is the most common form of diabetes among children and adolescents [2]. According to estimates by the International Diabetes Federation (2021), 1.2 million children and adolescents up to 19 years old have type 1 diabetes globally [3]. In China, the incidence of T1DM in children was 1.93/100,000, with an increase of 6.5% per year during 2010–2013[4]. Meanwhile, in recent years, type 2 DM (T2DM) has been increasingly diagnosed among children and adolescents. In America, the incidence of T2DM increased from 9.0/100,000 in 2002–2003 to 13.8 in 2014–2015 among persons aged 10–19 years [5]. At present, the main goal of treatment with DM is glycemic control to reduce the occurrence and development of complications. The American Diabetes Association (ADA) recommended that the hemoglobin A1c (HbA1c) target for children and adolescents should be less than 7.5%. HbA1c ≥7.5% increased the risk of developing complications, such as severe hypoglycemia (SH) and diabetic ketoacidosis (DKA) [6, 7].

Depressive symptoms are common mental health problems in patients with diabetes among children and adolescents. A review showed that the pooled prevalence of depressive symptoms was 30.04% among children and adolescents with T1DM [8]. The prevalence rate of depressive symptoms with diabetes is two to three times higher than the general population [9]. There exists inconsistenty between depressive symptoms and glycemic control. Mild or more serious depressive symptoms among adolescents with type 1 diabetes were associated with an 18% increased risk of poor glycemic control [10]. Some results also showed the depressive symptoms were positively related with the HbA1c, and were an important predictor of HbA1c change [11, 12]. On the contrary, the results of a study in New Zealand in adolescents with diabetes indicated that there are no association between depressive symptoms and glycemic control [13]. Therefore, the relationship between depressive symptoms and glycemic control needs to be further studied.

In those evidence supporting depressive symptoms with higher HbA1c, diabetes self-care behaviors were suspected to play a role between them. Diabetes self-care behaviors refer to the daily health-related activities, such as management of blood glucose, insulin administration, physical activity, dietary habits, and so on. Schmitt et al. [14] found the more severe the depressive symptoms were, the worse the self-management behaviors of patients with diabetes possessed. Similarly, a review found depression negatively correlated with diet control and physical exercise [15].In terms of the relationship between diabetes self-care and blood glycemic control, a longitudinal descriptive study showed that diabetes self-care activities were associated with worse metabolic control [16].Then, intervention studies suggest that the psychological status and blood glucose of patients with diabetes who received self-management education were significantly improved compared to control group received routine outpatient education [17].Through education, the psychological status of patients with diabetes improved, and blood glucose could be better controlled.

Few studies evaluate the mediation effect of self-care behaviors between depressive symptoms and glycemic control. In the United States, a prospective study including 998 middle-aged and older adults with T2DM demonstrated that health-related behaviors accounted for 13% of the association between depressive symptoms and glycemic control [18]. However, that study relied on middle-aged and older adults in patients with T2DM, rather than the population of children and adolescents. To our knowledge, self-care behaviors mediated the relationship between depressive symptoms and glycemic control among children and adolescents in China with DM patients has been not investigated. Thus, the goal of the present study is to examine the associations between depressive symptoms and glycemic control among children and adolescents with diabetes between the age of 8–18 years old. In addition, we aimed to examine the mediating role of self-care behaviors in this association.

## 2. Materials and Methods

### 2.1. Participants

This was a cross-sectional study of children and adolescents with diabetes between the age of 8 and 18 years old who were enrolled in four Hospital of China (the First Affiliated Hospital of Anhui Medical University, the Second Affiliated Hospital of Anhui Medical University, Anhui Children's Hospital, and Anqing Municipal Hospital) from May to September 2023. Inclusion criteria included: T1DM or T2DM diagnosed based on the WHO diagnostic criteria for diabetes, 8–18 years old, diabetes duration ≥ 6 months, ability of reading and writing Chinese and communicating effectively in Chinese. Exclusion criteria included: disabilities, mental illness, serious underlying medical conditions, and those who could not complete the investigation. Patients and their families were informed about the purpose and procedure of the study and voluntarily signed an informed consent form (minors will be signed by their guardians on their behalf). The investigator supervised and guided the questionnaire filling process throughout. At the end of the questionnaire survey, the investigator transcribed the latest biochemical test indexes in the patients' cases on the spot. A total of 215 questionnaires were collected in this study, and 207 questionnaires were included in the statistical analysis after removing the questionnaires that lacked important research information.

This study obtained ethical approval from Anhui Medical University, ID: 83230100. Details of the Strengthening the Reporting of Observational Studies in Epidemiology (STROBE) for this study are available in the Supporting Information.

### 2.2. Measurements

The questionnaire consists of five modules: general demographic characteristics, diabetes-related information, depressive symptoms, self-care behaviors, and glycemic control. The Chinese version of the Children's Depression Inventory (CDI) was used to assess the depressive symptoms in the last 2 weeks [19]. The scale consists of anhedonia (eight items), negative emotion (seven items), low self-esteem (four items), low efficacy (four items), and interpersonal relationship (four items), with a total of 27 items. Each item is rated on a scale of 0–2 (e.g., 0 = I occasionally feel like crying; 1 = I often feel like crying; 2 = I feel like crying every day). The total score ranges from 0 to 54. Higher scores indicated more severe depressive symptoms, and ≥19 score was thought to have depressive symptoms [20]. Cronbach's α was 0.872 in this study.

The Chinese version of the Summary of Diabetes of Self-Care Activities (SDSCA) was used to evaluate the level of diabetes self-care behaviors in children and adolescents with DM in the past 1 week. The scale has a total of 11 items, mainly including six dimensions, ordinary diet (two items), special diet (two items), exercise (two items), blood glucose monitoring (two items), foot care (two items), and medication as advised by the doctor (one item). Each item was asked how many days they had completed in the past 7 days, with scores ranging from 0 to 7. The total score ranges from 0 to 77. The higher the score indicated the better of self-care [21]. The reliability and validity of the scale were good in children and adolescents with DM, and Cronbach's α was 0.836 in this study.

The proportion of HbA1c to the total amount of hemoglobin can reflect the average blood glucose level of diabetic patients in the first 2–3 months (the average life span of red blood cells). At present, HbA1c is the only long-term blood glucose control indicator with reliable outcome data. The higher the percentage of HbA1c indicated the worse blood glucose level of patients in the recent 2–3 months. The values of HbA1c of children and adolescents with diabetes were obtained from patients' medical history cases or self-reporting.

### 2.3. Statistical Analysis

Mean ± standard deviation (SD) was used to describe continuous variables, and count (proportion) was expressed for categorical variables. The *t*-test, analysis of variance (ANOVA) were conducted to clarify the differences of glycemic control level among children and adolescents with diabetes on demographic characteristics, diabetes-related information. The relationships between depressive symptoms, self-care behaviors, and glycemic control were assessed using the Pearson correlation analysis. A structural equation model was used to assess the relationship between depressive symptoms and HbA1c with self-care behaviors as the latent variable. The bootstrap was used to test the direct effects and indirect effects of the self-care behaviors by using bootstrapping with 5000 bootstrap samples. All analyses were performed using SPSS 25.0 and AMOS 24.0.

## 3. Results

### 3.1. Sample Characteristics

A total of 215 children and adolescents with diabetes participated, but eight cases were excluded from the analyses for essential missing data. The final sample of 207 individuals included 95 females and 112 males, with 42.5% participants were 8∼12 years old. 25.6% participants were overweight or obesity and 34.8% participants were the only one child without siblings. 92.3% participants were in school, and the most (90.8%) participants' primary caregiver were parents. The participants of the household monthly income <4000 yuan accounted for 34.8%. 56 patients (27.1%) were treated strictly from their guardians. 79.7% participants had T1DM and 29.0% participants had a family history of diabetes. 11 participants' mothers had gestational diabetes at the time of pregnancy. 175 participants' diabetes duration was <5 years. The proportion of patients receiving nondrug therapy (exercise and diet), only oral drug therapy, only insulin therapy and oral drug plus insulin therapy were 5.8%, 3.9%, 72.5%, and 17.9%, respectively. 17.9% participants had diabetic complications. 130 participants (62.8%) reported they were familiar with diabetes knowledge. 151 participants (72.9%) had less than four return visits per year due to diabetes. The total score of depressive symptoms was 12.71 ± 6.73.The total score of self-care behaviors was 42.31 ± 14.09, and the scores of ordinary diets, special diets, exercise, blood glucose monitoring, foot care, and prescribed medication were 8.84 ± 4.78, 6.82 ± 3.09, 5.91 ± 3.82, 7.37 ± 4.76, 5.95 ± 5.04, and 5.06 ± 2.09, respectively. The HbA1c of the patients was 9.14 ± 2.55%. All the sample characteristics were shown in Table 1

### 3.2. Comparison of Participants' Characteristics With HbA1c

The HbA1c values were found to be higher in participants aged 12–18 years and overweight or obesity than their counterparts (9.78 ± 2.72 vs. 8.27 ± 2.02, *p*  < 0.001 and 9.39 ± 2.66 vs. 8.42 ± 2.07, *p*  < 0.05, respectively). The patients of dropping out of school or in middle school had higher HbA1c values than in primary school and college (*p* < 0.05). Those children with a household monthly income of less than 4000 yuan had the highest HbA1c (10.26 ± 2.66) compared to other groups (*p* < 0.001). When the primary caregiver was grandparents or others, the children had higher HbA1c relative to parents as caregivers (10.34 ± 3.36 vs. 9.02 ± 2.43, *p*  < 0.05). T2DM patients' HbA1c values were higher than T1DM and those with no genetic history of diabetes' value was higher than having genetic history of diabetes (10.36 ± 2.95 vs. 8.83 ± 2.35, *p*  < 0.001 and 10.14 ± 2.74 vs. 8.72 ± 2.36, *p*  < 0.001, respectively). HbA1c of those patients receiving oral medication only or insulin therapy only were lower than those with nondrug therapy or oral medication together with insulin therapy (*p*  < 0.05), Those patients possessing complications and unfamiliar with diabetes knowledge showed higher HbA1c values than those without complications and familiar with diabetes knowledge (10.51 ± 2.94 vs. 8.83 ± 2.36, *p*  < 0.001 and 10.35 ± 2.84 vs. 8.41 ± 2.06, *p*  < 0.001, respectively). The details were shown in Table 2.

### 3.3. Correlation Analysis of Depressive Symptom, Self-Care Behaviors, and Glycemic Control

The Pearson correlation analysis was conducted on HbA1c, depressive symptom score, self-care behaviors score, and six dimensions of self-care behaviors, as well in DM children and adolescents (Table 3). The results showed that the depressive symptom score was positively correlated with HbA1c (*r* = 0.32). The scores of depressive symptoms were negatively correlated with the total score of self-care behaviors (*r* = −0.28), general diet (*r* = −0.18), blood glucose monitoring (*r* = −0.17), and foot care (*r* = −0.23). HbA1c was negatively correlated with total score of self-care behaviors (*r* = −0.39), general diet score (*r* = −0.25), exercise score (*r* = −0.18), blood glucose monitoring score (*r* = −0.31), foot care score (*r* = −0.25), and medication compliance score (*r* = −0.21).

### 3.4. Mediating Effect of Self-Care Behaviors on the Relationship Between Depressive Symptoms and Glycemic Control

**Figure 1:**
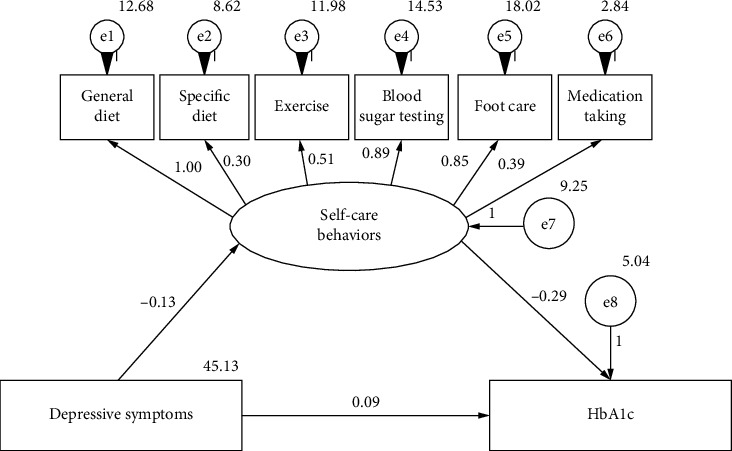
The SEM for the relationship between depressive symptoms and HbA1c among patients with DM.

The structural equation model of the relationship and the path coefficient between depressive symptoms and glycemic control are shown in Figure 1. Depressive symptoms are negatively correlated with self-care behaviors, and the standardized path coefficient *β* value is −0.133 (t = −3.404, *p*  < 0.001). Self-care behaviors are negatively correlated with HbA1c and standardized path coefficient *β* is −0.287, (*t* = 0.069, *p*  < 0.001). Depressive symptoms were positively correlated with HbA1c and standardized path coefficient *β* is 0.086, (*t* = 3.378, *p*  < 0.001). The structural equation model of the relationship between depressive symptoms and blood glucose control established by self-care behaviors as a latent variable is shown as follows: *χ*^2^/DF = 0.703, RMSEA < 0.001, GFI = 0.984, AGFI = 0.971, NFI = 0.943, CFI = 1.000, IFI = 1.000, the fit of the model was good and acceptable.

The mediating effect test results of this model showed that the direct effect of the depressive symptoms and glycemic control (HbA1c) pathway was 0.086 (95%CI: 0.038, 0.137), *p*  < 0.001, the indirect effect was 0.038, 95%CI (0.016, 0.071), *p*  < 0.001 and the total effect was 0.124, 95%CI (0.073, 0.175), *p*  < 0.001.

### 3.5. Sensitivity Analysis

To comprehensively assess the robustness of the mediating effect model in our study, we initially conducted a meticulous examination of the mediating effect solely among patients with type 1 diabetes. Subsequently, patients with type 2 diabetes were incorporated into the analysis framework to perform a sensitivity analysis. The results (Table 4) showed that when only patients with type 1 diabetes were included, the coefficients of the mediating effect pathway were consistent with those of the combined analysis (direct effect: 8.6% vs. 9.6%; indirect effect: 3.8% vs. 3.8%), and the statistical significance remained unchanged (*p*  < 0.005).

## 4. Discussion

Consistent with previous findings, children and adolescents with diabetes have suboptimal glycaemic control. A multicentre study in China suggested that the average HbA1c in children was 9.5%, with a single-center HbA1c compliance rate of only 15% in China [22]. Another study conducted in the United States showed that only 20% of young diabetics between the ages of 13 and 18 maintained glycemic control at appropriate levels recommended by the ADA [23]. Recent studies have highlighted the long-term benefits of ensuring optimal glycaemic control during childhood and adolescence in preventing complications in adulthood [24]. Therefore, it is important to explore the risk factors behind poor glycaemic control, which can contribute to better functioning and management of the disease.

The results of descriptive analyses showed that HbA1c in children and adolescents with diabetes differed in terms of age, type of diabetes, BMI, therapy method, complications, diabetes education received, current education, household income, primary caregiver, and genetic history of diabetes. In the results of a meta analysis of risk factors for glycaemic control in children and adolescents with type 1 diabetes, age was not associated with HbA1c levels [25]. However, due to the risk of hypoglycemia, and the demands of growth and development, appropriately relax the glycaemic criteria for school-age children. This may partly explain why HbA1c is higher in 8–12 year olds than in 12–18 year olds. Obesity may be both a risk factor for T2DM and may also imply a higher HbA1c. A JAMA systematic review showed that the overall prevalence of obesity in pediatric patients with T2DM was 75.27%, implying that approximately 3/4 of pediatric patients with T2DM were also obese. It is the strong association between T2DM and obesity that may have contributed to the higher HbAlc in patients with T2DM in our results. Patients treated with oral medication only or insulin only had lower HbA1c than patients not treated with medication or treated with oral medication with insulin. This may be due to the fact that patients who only adopted dietary control or exercise methods were unable to effectively control their blood sugar levels, and that patients undergoing combination therapy had more severe conditions [26]. The relationship between complications and glycaemic control may be bidirectional [27, 28], so that participants with complications had a higher HbA1c than those without complications. Knowledge of diabetes facilitates patients' glycaemic control. Patients who are knowledgeable about diabetes are able to develop a practical ability to control their blood glucose [29].

We also found that stress from the family and study environment may contribute to poorer glycaemic control. Previous studies have shown that lower socioeconomic status, not living with biological parents, nonbiparental families, and family barriers increase HbA1c levels in children and adolescents with diabetes [25]. This is consistent with our findings. Low-income families may have difficulty affording resources such as high-quality glucose-lowering medications, ambulatory glucose meters, and other resources, and may have difficulty maintaining a healthy lifestyle due to financial constraints. Families with a genetic history may place more emphasis on diabetes-related care for their offspring and take better care of their patients with better glycaemic control. Having biological parents who are not primary caregivers may weaken the continuity of diabetes management, with a lack of stable caregivers to oversee diet, medication, and glucose monitoring [30]. Second, child and adolescent patients whose parents are primary caregivers may be more direct and natural in expressing their emotions and needs. On the other hand, patients who do not live with their biological parents are influenced by traditional Chinese concepts and are afraid of being criticized for “not sensible,” and are more likely to hide their true emotions and lack the initiative to express their needs when they are aggrieved or angry. This may lead to a build-up of emotions in the long term, such as sudden outbursts of temper or reticence. Patients who dropped out of school or in middle school had higher HbA1c values than those in primary school and university. This may stem from three factors: academic pressure on high academic achievement and strict teaching management may affect glycaemic control. Teachers who are not sufficiently aware of the disease may ignore patients' monitoring needs for “fear of affecting academic performance," such as denying them the use of mobile phones and ambulatory glucose meters in the classroom. In terms of peer understanding, junior high school students are in a sensitive period of peer relationships, and their classmates have limited knowledge of diabetes, and may alienate the patient due to “fear of trouble.” If the teacher also lacks understanding, fails to guide the classroom atmosphere in a timely manner, or even acquiesces to such isolation, the patient may be plunged into even deeper loneliness. In terms of stigma, the fear of being “different” is even stronger: the need to take insulin injections and monitor blood glucose at school may result in a sense of shame due to the fear of being talked about by classmates, leading to a deliberate avoidance of management behaviors.

These factors intertwine to form a complex network of influences that may exacerbate depressive mood in stages. Several previous studies have assessed the relationship of depressive symptoms and glycemic control, however there is heterogeneity in reported studies. McClintock et al. [13] thought that diabetes in adolescents and youth with depressive symptoms was not associated with glycemic control. However, some other studies showed adolescent patients with diabetes who had depressive symptoms displayed higher HbA1c values than those without depressive symptoms peers [10, 31]. Our study found that depressive symptoms were positively correlated with HbA1c. There may be several mechanisms as follows linking depressive symptoms and glycemic control in children and adolescents with DM. Mental disorders, such as anxiety, which is significantly associated with glycemic control. Previous studies showed children and adolescents with diabetes and anxiety disorders had worse glycemic control, compared to those without anxiety disorders [32]. Depressive and anxiety symptoms are often comorbid. Elevated depressive symptoms also experience symptoms of anxiety [33]. Comorbid depressive symptoms and anxiety disorders with diabetes may increase the higher risk of worse glycemic control. Meanwhile, patients with depressive symptoms are prone to cutting physical activity, developing to sleep disorder, diet disorder and poor treatment compliance as well, which could affect glycemic control [34–36]. Moreover, self-care behaviors played a part mediating role in the association of depressive symptoms with glycemic control. This study shows that the total effect of depression on HbA1c in children and adolescents with diabetes is *β* = 0.124 (*p*  < 0.001). The direct effect is *β* = 0.086 (*p*  < 0.001), and the mediating effect through “self-care behavior” is *β* = 0.038 (*p*  < 0.001), accounting for 30.65% of the total effect. Although the mediation effect size did not reach the level of a “strong association,” this pathway suggests that diabetes management should not only focus on HbA1c but also attach importance to depressive symptoms and self-care behaviors. There are several mechanisms which may support the mediation effect of self-care behaviors between depressive symptom and glycemic control. One study reported that depressive symptoms were associated with one dimension of self-care behaviors-diet disorder in DM patients and diet disorder can lead to inflammation and immune responses, which increased the risk of DM and diabetes prognosis [37, 38]. McGrady et al. [39] demonstrated that another dimension of self-care behaviors-blood glucose monitoring partially mediated the association between depressive symptoms and glycemic control which is similar with our result [40]. Many diabetes patients with depressive symptoms took blood glucose testing when they were suffering from treatment or feeling uncomfortable. They had poor compliance of patients with blood glucose monitoring which affected the patient's blood glucose control. Despite the effect of this single pathway is small, long-term depression can inhibit the self-care behaviors of children and adolescents, forming a vicious cycle where poor mood leads to lax management, which in turn results in poor blood glucose levels, and ultimately exacerbates the poor mood. Especially during the critical period of development, this not only affects current blood glucose levels but also solidifies unhealthy behavioral patterns, increasing the difficulty of metabolic control and the risk of psychological problems in adulthood. Therefore, early clinical identification and intervention of this associated pathway are of great significance for reducing long-term health damage.

There were several limitations to the present study. First, this study is a cross-sectional survey, which can only confirm the association of depressive symptoms and glycemic control among children and adolescents with DM. A longitudinal study to detect the present findings is needed. Second, although the sensitivity analysis showed that the model was robust, the excessively small sample size might lead to deviations or distortions in the results, making it difficult to reflect the real association. Therefore, the relevant conclusions need to be interpreted with caution, and future studies should expand the sample size for verification. Third, most of the information obtained in this study comes from electronic questionnaires, not verified by medical records. Finally, as in most studies, this study failed to investigate all confounders, such as blood glucose monitoring methods and insulin injection methods.

## 5. Conclusions

This study offers preliminary insights into the associations between psychological status, behavioral patterns, and metabolic control among children and adolescents with diabetes. It implies that in clinical practice, prioritizing depressive symptoms in this demographic and implementing targeted interventions to enhance their self-care capabilities may exert a favorable influence on optimizing glycemic management. Future investigations, including prospective cohort studies and interventional trials, are warranted to further validate the causal relationships between variables and the stability of the mediating pathways, thus furnishing practical evidence for improving health outcomes in this population.

## Figures and Tables

**Table 1:** The sociodemographic characteristics and diabetes related information among children and adolescents with DM.

Variable	*n*	%	Mean (SD)
Age (years)			
8∼12	88	42.5	—
Gender			
Female	95	45.9	—
BMI (kg/m^2^)			
Overweight/obesity	53	25.6	—
Only one child without siblings			
Yes	72	34.8	—
Current education			
Drop out	16	7.7	—
Primary school	88	42.5	—
Middle school	91	44.0	—
College	12	5.8	—
Household monthly income (CNY)			
<4000	72	34.8	—
4000∼8000	75	36.2	—
8000∼12000	36	17.4	—
>12000	24	11.6	—
Father's education level			
Primary school or below	24	11.5	—
Junior high school	104	50.2	—
Senior high school	50	24.2	—
College or above	29	14.0	—
Mother's education level			
Primary school or below	48	23.2	—
Junior high school	89	43.0	—
Senior high school	38	18.4	—
College or above	32	15.5	—
The primary caregiver			
Parents	188	90.8	—
Grandparents or whatever	19	9.2	—
Parenting style			
Strict	56	27.1	—
Democratic	110	53.1	—
Others	41	19.8	—
Type of diabetes			
T1DM	165	79.7	—
Genetic history of diabetes			
Yes	60	29.0	—
Maternal history of gestational diabetes			
Yes	11	5.3	—
Diabetes duration(years)			
<5	175	84.5	—
Therapy method			
Nondrug therapy	12	5.8	—
Oral only medication	8	3.9	—
Insulin only therapy	150	72.5	—
Oral medication and insulin therapy	37	17.9	—
Complication			
Yes	37	17.9	—
Be familiar with diabetes			
Yes	130	62.8	—
Number of diabetes return visits per year			
<4	151	72.9	—
Depressive symptoms	—	—	12.71 (6.73)
Self-care behaviors (SDSCA total)	—	—	42.31 (14.09)
General diet	—	—	8.84 (4.78)
Specific diet	—	—	6.82 (3.09)
Exercise	—	—	5.91 (3.82)
Blood sugar testing	—	—	7.37 (4.76)
Foot care	—	—	5.95 (5.04)
Medication taking	—	—	5.06 (2.09)
HbA1c	—	—	9.14 (2.55)

**Table 2:** Comparison of HbA1c in sociodemographic profile and diabetes related information of patients with DM.

Variable	HbA1c (%)		
	Mean (SD)	t/*F*	*P*
Age (years)	—	−4.384	<0.001
8∼12	8.27 (2.02)	—	—
12∼18	9.78 (2.72)	—	—
Gender	—	0.320	0.750
Male	9.19 (2.76)	—	—
Female	9.08(2.31)	—	—
BMI (kg/m^2^)	—	2.411	0.017
Normal	8.42 (2.07)	—	—
Overweight/obesity	9.39 (2.66)	—	—
Only one child without siblings	—	1.621	0.107
Yes	9.35 (2.57)	—	—
No	8.75 (2.49)	—	—
Current education	—	5.207	0.002
Drop out	9.49 (2.35)	—	—
Primary school	8.39 (2.14)	—	—
Middle school	9.82 (2.77)	—	—
College	8.93 (2.62)	—	—
Household monthly income (CNY)	—	11.465	<0.001
<4000	10.26 (2.66)	—	—
4000∼8000	8.85 (2.24)	—	—
8000∼12000	7.49 (1.83)	—	—
>12000	9.15 (2.59)	—	—
Father's education level	—	2.043	0.109
Primary school or below	9.02 (2.52)	—	—
Junior high school	9.79 (2.55)	—	—
Senior high school	8.55 (2.64)	—	—
College or above	8.97 (2.46)	—	—
Mother's education level	—	2.064	0.106
Primary school or below	9.43 (2.22)	—	—
Junior high school	9.05 (2.55)	—	—
Senior high school	9.69 (2.93)	—	—
College or above	8.29 (2.42)	—	—
The primary caregiver	—	−2.163	0.032
Parents	9.02 (2.43)	—	—
Grandparents or other	10.34 (3.36)	—	—
Parenting style	—	0.549	0.578
Strict	8.98 (2.53)	—	—
Democratic	9.09 (2.52)	—	—
Others	9.50 (2.70)	—	—
Type of diabetes	—	−3.572	<0.001
T1DM	8.83 (2.35)	—	—
T2DM	10.36 (2.95)	—	—
Genetic history of diabetes	—	−3.731	<0.001
Yes	8.72 (2.36)	—	—
No	10.14 (2.74)	—	—
Maternal history of gestational diabetes	—	0.730	0.466
Yes	9.17 (2.59)	—	—
No	8.59 (1.89)	—	—
Diabetes duration (years)	—	−0.573	0.566
<5	9.09 (2.50)	—	—
≧5	9.37 (2.87)	—	—
Therapy method	—	3.596	0.015
Nondrug therapy	10.36 (3.15)	—	—
Oral medication only	8.61 (2.80)	—	—
Insulin therapy only	8.83 (2.34)	—	—
Oral medication and insulin therapy	10.09 (2.84)	—	—
Complication	—	−3.729	<0.001
Yes	10.51 (2.94)	—	—
No	8.83 (2.36)	—	—
Be familiar with diabetes	—	—	—
Yes	8.41 (2.06)	5.670	<0.001
No	10.35 (2.84)	—	—
Number of diabetes follow-up visits per year	—	0.845	0.394
<4	9.23 (2.61)	—	—
≧4	8.88 (2.38)	—	—

**Table 3:** Correlation analysis of HbA1c, depressive symptoms, and self-care behaviors among children and adolescents with DM (*r*).

Variable	1	2	3	4	5	6	7	8	9
1. Depressive symptoms	1.00	—	—	—	—	—	—	—	—
2. HbA1c	0.32*⁣*^*∗∗*^	1.00	—	—	—	—	—	—	—
3. Self-care behaviors (SDSCA total)	−0.28*⁣*^*∗∗*^	−0.39*⁣*^*∗∗*^	1.00	—	—	—	—	—	—
4. General diet	−0.18*⁣*^*∗*^	−0.25*⁣*^*∗∗*^	0.71*⁣*^*∗∗*^	1.00	—	—	—	—	—
5. Specific diet	−0.12	−0.10	0.40*⁣*^*∗∗*^	0.27*⁣*^*∗∗*^	1.00	—	—	—	—
6. Exercise	−0.09	−0.18*⁣*^*∗∗*^	0.52*⁣*^*∗∗*^	0.28*⁣*^*∗∗*^	0.08	1.00	—	—	—
7. Blood sugar testing	−0.17*⁣*^*∗*^	−0.31*⁣*^*∗∗*^	0.68*⁣*^*∗∗*^	0.36*⁣*^*∗∗*^	0.23*⁣*^*∗∗*^	0.25*⁣*^*∗∗*^	1.00	—	—
8. Foot care	−0.23*⁣*^*∗∗*^	−0.25*⁣*^*∗∗*^	0.67*⁣*^*∗∗*^	0.37*⁣*^*∗∗*^	0.08	0.21*⁣*^*∗∗*^	0.30*⁣*^*∗∗*^	1.00	—
9. Medication taking	−0.11	−0.21*⁣*^*∗∗*^	0.61*⁣*^*∗∗*^	0.40*⁣*^*∗∗*^	0.15*⁣*^*∗*^	0.28*⁣*^*∗∗*^	0.35*⁣*^*∗∗*^	0.32*⁣*^*∗∗*^	1.00

*⁣* ^*∗*^ *p* < 0.05.

*⁣* ^*∗∗*^ *p* < 0.01.

**Table 4:** Comparison of mediation effect results between combined diabetes types and type 1 diabetes only.

Type of analysis	Parameter	Estimate	Lower	Upper	*p*-Value
Mediation results for combined effects	Indirect effect	0.038	0.016	0.071	0.001
	Direct effect	0.086	0.038	0.137	0.001
	Total effect	0.124	0.073	0.175	<0.001

Mediation results for type 1 diabetes only	Indirect effect	0.038	0.011	0.076	0.003
	Direct effect	0.097	0.044	0.154	0.001
	Total effect	0.134	0.084	0.188	<0.001

## Data Availability

The data are not publicly available due to privacy or ethical restrictions.

## Supporting Information

Supporting Information The Supporting Information of this study includes a Research Guidelines List, which is formulated in strict accordance with the STROBE-checklist-v4-cross-sectional.
